# VEXAS-Syndrom

**DOI:** 10.1007/s00393-022-01169-6

**Published:** 2022-02-18

**Authors:** M. Zeeck, I. Kötter, M. Krusche

**Affiliations:** 1 Sektion für Rheumatologie und Entzündliche Systemerkrankungen, Universitätsklinikum Hamburg-Eppendorf (UKE), Martinistr. 52, 20246 Hamburg, Deutschland; 2 Klinik für Rheumatologie und Immunologie, Bad Bramstedt, Deutschland

**Keywords:** Autoinflammation, Vakuolen, Somatische Mutation, Myelodysplastisches Syndrom, Polychondritis, Autoinflammation, Vacuoles, Somatic mutation, Myelodysplastic syndrome, Polychondritis

## Abstract

Das VEXAS-Syndrom ist eine neu identifizierte autoinflammatorische Systemerkrankung. Das Akronym VEXAS steht hier für *V*acuoles, *E*1 enzyme, *X*-linked, *A*utoinflammatory, *S*omatic. Die Erkrankung beruht auf einer erworbenen somatischen Mutation des *UBA1*-Gens. Dieses kodiert für das E1-Enzym, welches wiederum für die Ubiquitinierung von Proteinen verantwortlich ist. Aufgrund der Lage des Gens auf dem X‑Chromosom betrifft die Erkrankung überwiegend Männer (in der zweiten Lebenshälfte). Die Patienten weisen eine Plethora an inflammatorischen klinischen Symptomen – oft mit Überlappung von hämatologischen, dermatologischen und rheumatologischen Krankheitsbildern – auf. Insbesondere das Vorliegen von zytoplasmatischen Vakuolen im Knochenmark ist charakteristisch. In dieser Arbeit berichten wir über den klinischen Fall eines VEXAS-Patienten und geben einen Überblick über die Pathophysiologie, Klinik und Diagnostik des Erkrankungsbildes.

## Abstract (en)

The VEXAS syndrome is a recently identified autoinflammatory systemic disease. The acronym VEXAS stands for Vacuoles, E1 enzyme, X‑linked, Autoinflammatory, Somatic. The disease is due to an acquired somatic mutation of the *UBA1 *gene, which encodes for the E‑1 enzyme, which in turn is responsible for the ubiquitination of proteins. Due to its location on the X chromosome, the disease predominantly affects men (in the second half of life). The patients present with a plethora of inflammatory clinical symptoms, often with overlap of hematologic, dermatologic, and rheumatologic syndromes. In particular, the presence of cytoplasmic vacuoles in the bone marrow is characteristic. In this article we report the clinical case of a VEXAS patient and give an overview of the pathophysiology, clinical symptoms and diagnostics of the disease.

Im Jahr 1999 wurde von der Arbeitsgruppe um Dan Kastner erstmals das Konzept der Autoinflammation beschrieben [1]. Dies umfasst eine pathogene Entzündung, welche unabhängig von Autoantigenen zu einer Hyperaktivierung des Immunsystems mit Inflammationsreaktion führt.

In den darauffolgenden Jahren konnte eine Differenzierung von monogenetischen autoinflammatorischen Erkrankungen (wie z. B. das familiäre Mittelmeerfieber oder das Cryopyrin-assoziierte periodische Syndrom) und polygenetischen Erkrankungen (wie z. B. der Morbus Still) erfolgen. Überwiegend manifestieren sich diese Erkrankungen erstmalig im Kindes- oder jungen Erwachsenenalter.

Interessanterweise tritt jedoch ein Teil der autoinflammatorischen Erkrankungen auch in Assoziation mit hämatologischen Veränderungen oder Erkrankungsbildern bei älteren Patienten auf. Prominentestes Beispiel ist das Schnitzler-Syndrom, welches gekennzeichnet ist durch eine monoklonale Hypergammaglobulinämie vom Typ IgM oder IgG sowie urtikarielles Exanthem, Fieber, neutrophile Dermatitis, Knochenveränderungen und systemische serologische Inflammationszeichen [2]. Weiterhin sind z. B. auch für das myelodysplastische Syndrom (MDS) hyperinflammatorische Immunphänomene beschrieben [3], sodass aktuell sogar der Begriff der „Hämatoinflammation“ in diesem Zusammenhang eingeführt wurde [4].

In diesem Kontext beschrieben Beck et al. 2020 erstmalig im *New England Journal of Medicine* eine neue autoinflammatorische Erkrankung, die sie VEXAS-Syndrom nannten. Die durch eine somatische Mutation erworbene Erkrankung tritt ebenfalls erst im fortgeschrittenen Erwachsenenalter auf und weist mehrere hämatoinflammatorische Features auf [5]. Das Akronym VEXAS steht hier für *V*acuoles, *E*1 enzyme, *X*-linked, *A*utoinflammatory, *S*omatic.

## Genetik und Pathophysiologie

In der Erstbeschreibung des VEXAS-Syndroms konnten Beck at al. 25 Männer identifizieren, welche eine somatische Missense-Mutation des Codons 41, des sich auf dem X‑Chromosom befindlichen *UBA1-Gens*, aufwiesen. Hierbei liegt eine pathogene Mosaikmutation im Gen vor. Mittlerweile wurden auch weitere Mutationen im *UBA1-Gen* außerhalb des Codons beschrieben [6, 7]. Das *UBA1-Gen* kodiert für das E1-Enzym, das essenziell für die Initiierung der Ubiquitinierung von Proteinen ist. In mehr als der Hälfte der Patienten konnte in den hämatopoetischen Stammzellen diese Mutation nachgewiesen werden. Diese betraf insbesondere die myeloischen Zellreihe. Mittels weiterführender Transkriptionsanalysen konnte gezeigt werden, dass bei den betroffenen Patienten die entsprechenden Zellreihen im peripheren Blut eine verringerte Ubiquitinierung aufwiesen, was zur Aktivierung von Signalwegen des angeborenen Immunsystems führte. Dies wiederum initiiert die Aktivierung von Signalwegen für die Synthese von Interleukin‑1, Interleukin‑6, Interleukin‑8, Interferon‑γ und Tumor-Nekrose-Faktor α.

## Klinisches Bild

Das VEXAS-Syndrom tritt im fortgeschrittenen Erwachsenenalter auf (medianes Manifestationsalter 64 Jahre) und ist u. a. gekennzeichnet durch rezidivierende Fieberschübe sowie deutlich erhöhte serologische Entzündungszeichen [5]. Die Patienten weisen eine Plethora an klinischen Symptomen auf, wobei durchaus Überlappungen mit verschiedenen rheumatologischen, dermatologischen und insbesondere auch hämatologischen Erkrankungen bestehen.

Hämatologische Veränderungen lassen sich bei einer Vielzahl der Patienten feststellen. Insbesondere eine makrozytäre Anämie sowie eine Thrombopenie sind charakteristisch. Häufig findet sich bei den Patienten das Vollbild eines MDS (31 %). Außerdem wurde auch das Auftreten einer monoklonalen Gammopathie unklarer Signifikanz (MGUS) (7 %) oder ein Myelom (1 %) berichtet [8].

Weiterhin treten bei einem Großteil der Patienten dermatologische Veränderungen auf. Hierzu zählen u. a. eine neutrophile Dermatitis, schmerzhafte Papeln [9], Erythema nodosum [10], eine kutane Vaskulitis der kleinen und mittleren Gefäße sowie febrile Dermatose (Sweet-Syndrom) [5].

Darüber hinaus zeigt das VEXAS-Syndrom auch eine Überlappung mit verschiedenen rheumatologischen Krankheitsbildern: Besonders das Auftreten der rezidivierenden Polychondritis (RP) wird vermehrt berichtet [11, 12]. In der VEXAS-Kohorte von Beck et al. hatten 60 % der Patienten eine RP. In einer weiteren Analyse konnten Ferrada et al. in der Analyse einer RP-Kohorte von 92 Patienten bei 7 Patienten (7,6 %) eine UBA1-Mutation nachweisen. Interessanterweise hatten alle VEXAS-RP-Patienten eine Mitbeteiligung des Nasen- und Ohrenknorpels, jedoch keine Chondritis der Rippen oder der Atemwege [11]. Darüber hinaus wurde auch das Vorkommen bei Vaskulitiden wie der ANCA-assoziierten Vaskulitis [13], Polyarteriitis nodosa oder Großgefäßvaskulitis berichtet [5].

**Figure 1:**
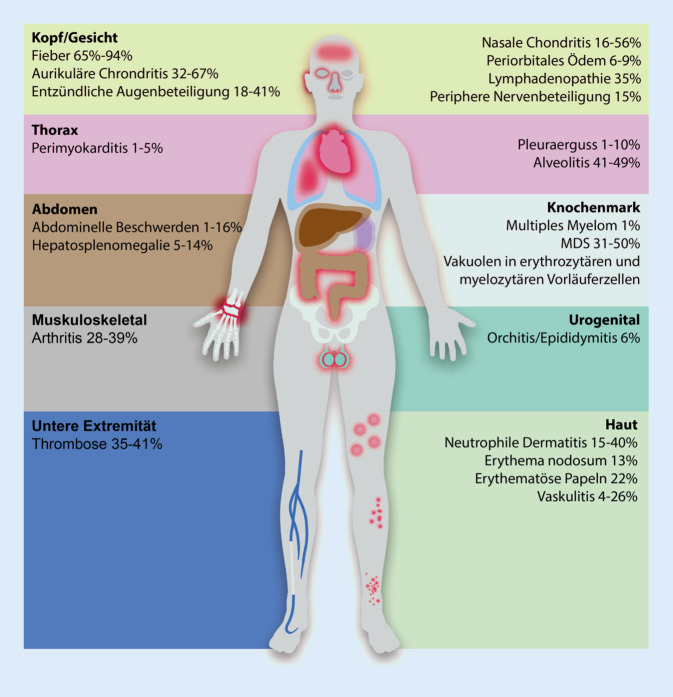

Des Weiteren finden sich bei einer Vielzahl der Patienten pulmonale Infiltrate mit Alveolitis (49 %). Ebenfalls treten bei bis zu 40 % der Patienten thromboembolische Komplikationen beschrieben (40 %) [8]. *Eine Übersicht über die klinischen Symptome zeigt *Abb. 1*.*

In einer französischen Analyse mit 116 Patienten konnten 3 Cluster identifiziert werden [14]:

1. MDS-bezogener Phänotyp nahe der Erstbeschreibung mit rezidivierendem Fieber, Chondritis und venöser Thromboembolie,
2. ein Cluster mit leichter bis mittelschwerer Erkrankung, geringerem Fieber, Chondritis und Thromboembolie,
3. ein „entzündliches“ Cluster, charakterisiert durch kutane Vaskulitisläsionen und hoher Rezidivrate.

Ebenfalls zeigen sich in der Arbeit erste Hinweise, dass es eine Assoziation zwischen Phänotyp-Genotyp gibt und sich hierdurch die Prognose verändert, hierfür bedarf es jedoch laut Aussage der Autoren weiterer Untersuchungen [14].

## Diagnostik und die Rolle des X-Chromosoms

Bei Patienten mit VEXAS-Syndrom sind die serologischen Entzündungszeichen meist stark erhöht. Weiterhin zeigen sich Blutbildveränderungen im Sinne einer makrozytären Anämie sowie eine Thrombopenie. In der Knochenmarkbiopsie finden sich darüber hinaus die charakteristischen zytoplasmatischen Vakuolen. Diese sind prädominant in den Promyelozyten, Myelozyten sowie den erythrozytären Vorläuferzellen und Blasten zu finden [15].

Obwohl es sich bei VEXAS im Wesentlichen um eine Erkrankung der hämatopoetischen Vorläuferzellen handelt, können Mutationen auch im peripheren Blut nachgewiesen werden, da es zu einer starken klonalen Expansion mutierter, zirkulierender myeloischer Zellen, nicht aber von T‑ oder B‑Lymphozyten kommt. Dies ist möglicherweise auf eine negative Selektion mutierter Lymphozyten im Knochenmark zurückzuführen. Zum Zeitpunkt der genetischen Diagnose wird fast immer eine hohe klonale Mutationslast beobachtet. Erste Daten zeigen bisher jedoch keine Korrelation zwischen dem Anteil der Variantenallele und der Krankheitsdauer oder dem phänotypischen Schweregrad [11]. Während der exakte Mutationszeitpunkt unbekannt ist, werden somatische Mutationen in UBA1 bei Patienten mit dem VEXAS-Syndrom wahrscheinlich erst später im Leben erworben, was mit der Beobachtung übereinstimmt, dass die klinischen Symptome erst im fünften Lebensjahrzehnt oder später auftreten. Die Mechanismen, die die klonale Expansion vorantreiben, und die Geschwindigkeit der Expansion sind derzeit nicht bekannt [16].

Mittels humangenetischer Diagnostik sollte auf die somatische Mutation des *UBA1-Gens* auf dem X‑Chromosom getestet werden. Beck et al. stellten in ihrer Erstbeschreibung des Krankheitsbildes die Hypothese auf, dass die Erkrankung nur Männer betreffen würde, da das „zweite X‑Chromosom bei Frauen eine Schutzwirkung hätte“. Allerdings konnte das VEXAS-Syndrom mittlerweile auch bei Frauen nachgewiesen werden. Terrier et al. konnten die entsprechende Mutation im *UBA1-Gen* ebenfalls bei 2 betroffenen Frauen nachweisen: Die Patientinnen hatten Fieber, Chondritis mit erhöhten Entzündungsparametern – in beiden Fällen lag eine X‑chromosomale Monosomie vor [17]. Für die genetische Diagnostik sollte weiterhin bedacht werden, dass in der Sanger-Diagnostik Mosaike teilweise übersehen werden können und ein zusätzliches Next Generation Sequencing die diagnostische Sicherheit zur Erfassung der Mosaikmutation verbessern kann.

## Therapie und Prognose

Die Erkrankung hat häufig einen sehr schwerwiegenden und komplikationsreichen Verlauf – in der Kohorte von Beck et al. verstarben 40 % der Patienten. Weiterhin wurde bei den Patienten vermehrt über thromboembolische Ereignisse, Alveolitis und Intensivaufenthalte berichtet. Darüber hinaus wurde in Einzelfallberichten das Auftreten eines Makrophagenaktivierungssyndroms [18, 19] und auch das Vorkommen einer AA-Amyloidose mit dialysepflichtigem Nierenversagen geschildert [20].

Ein Großteil der Patienten bedarf einer intensiven immunsuppressiven Therapie. Neben verschiedenen antiinflammatorischen Therapiekonzepten (z. B. Interleukin‑1, Interleukin‑6 oder JAK-Blockade) werden meist hohe Glukokortikoiddosen zur Krankheitskontrolle benötigt. Weiterhin gibt es erste kleine Fallserien, die bei einem Teil der VEXAS-Patienten mit MDS den Nutzen von Azacitidin nahelegen [21]. Etablierte Therapiekonzepte liegen aktuell noch nicht vor.

Diarra et al. berichten von einem 46-jährigen Patienten, bei dem retrospektiv mittels Mutationsanalyse die Diagnose VEXAS gestellt werden konnte und der initial bei therapierefraktärer Polyarteriitis nodosa und Sweet-Syndrom erfolgreich allogen stammzelltransplantiert wurde [22].

## Zusammenfassung

Zusammenfassend lässt sich festhalten, dass das VEXAS-Syndrom eine durch somatische Mutation des *UBA1-Gens* erworbene autoinflammatorische Erkrankung ist, welche als Mimic verschiedener rheumatologischer Erkrankungen auftreten kann. Insbesondere bei Überlappung inflammatorischer Krankheitsbilder/Symptome bei älteren Patienten sollte an das Vorliegen des VEXAS-Syndroms gedacht werden. Es besteht eine deutliche Dominanz des männlichen Geschlechtes, wobei jedoch in sehr seltenen Einzelfällen die Erkrankung auch bei Frauen auftreten kann.

Neben verschiedenen inflammatorischen klinischen Symptomen sind insbesondere in der Knochenmarkbiopsie das Vorliegen von zytoplasmatischen Vakuolen in der myeloischen und erythrozytären Reihe sowie eine makrozytäre Anämie und Thrombopenie mit erhöhten serologischen Entzündungszeichen suggestiv für die Erkrankung. Zur Diagnosesicherung ist eine humangenetische Testung des *UBA1-Gens* indiziert. Die Erkrankung ist mit einer Vielzahl an klinischen Komplikationen vergesellschaftet und weist eine hohe Mortalität auf. Aktuell bestehen noch keine etablierten antiinflammatorischen Therapiekonzepte. Meist werden hohe Dosen an Glukokortikoiden zur Inflammationskontrolle benötigt.

## Fallbericht

### Anamnese

Wir berichten über den Fall eines 76-jährigen Patienten mit in der Vorgeschichte seit Januar 2018 bestehender unklarer Inflammation sowie rezidivierenden Fieberepisoden bis 39,5 °C, progredienter Dyspnoe, Polyarthralgien, Bizytopenie sowie Schmerzen und Schwellung der Augen. Der Patient stellte sich erstmalig im Juli 2021 mit erneuter Fieberexazerbation (39,1 °C) und deutlich erhöhten serologischen Entzündungszeichen in unserer Notaufnahme vor.

In den bereits ausführlich erfolgten Voruntersuchungen war bisher ein Befundkomplex aus oben genannten Symptomen mit Alveolitis und Lungenfibrose mit respiratorischer Insuffizienz und 2 l-Heimsauerstoffbedarf, Polyarthritis der Knie‑, Kiefer‑, MCP- und Handgelenke sowie beidseitiger Uveitis anterior bekannt. In einer externen Knochenmarkpunktion war ebenfalls der Verdacht auf einen toxischen Knochenmarkschaden gestellt worden.

Im Vorfeld waren bereits Therapieversuche mit Methotrexat 20 mg s.c. (über 8 Monate) sowie Tocilizumab 162 mg s.c. (über 10 Monate) erfolglos gewesen. Über einen Zeitraum von über 2 Jahren war der Patient nur unter erhöhter Steroiddosis (> 12 mg/Tag) klinisch kontrollierbar.

**Figure 2:**
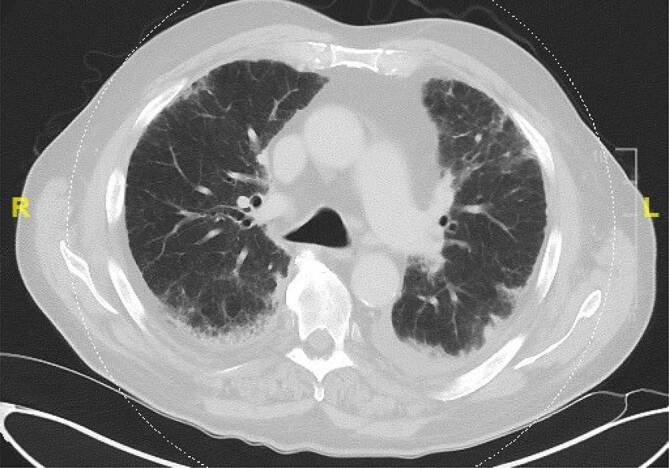

**Figure 3:**
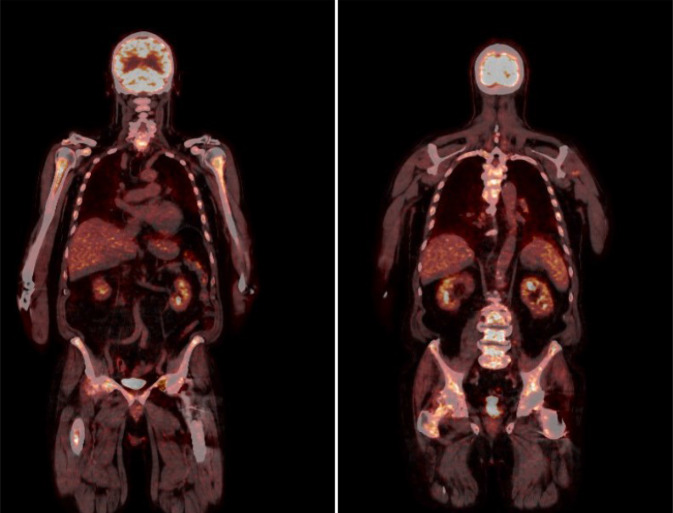

**Figure 4:**
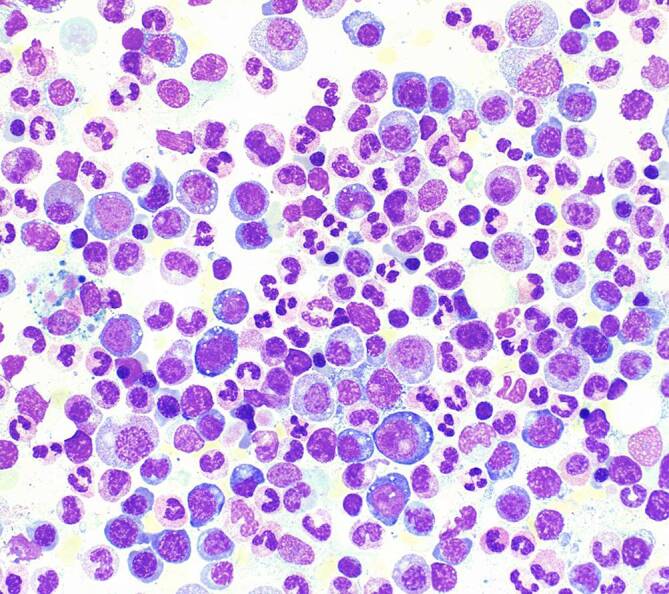

Laborchemisch imponierten bei Aufnahme ein deutlich erhöhtes CRP 302 mg/l (< 5 mg/l), eine makrozytäre Anämie HB 9,3 g/dl (14–17,5 g/dl), MCV 107fl (80–96/fl) l und eine Thrombozytopenie von 147 mrd/l (150–400 mrd/l). Die umfangreiche Erreger- und autoimmunserologische Diagnostik waren unauffällig. Im Thorax-CT sah man subpleurale, vorwiegend retikuläre Zeichnungsvermehrung mit Lungenfibrose und Milchglasinfiltraten. Weiterhin zeigte sich in der Bronchoskopie eine gemischt-lymphozytäre, eosinophile Alveolitis (Abb. 2). Im PET-CT sah man einen generalisierten deutlich erhöhten Knochenstoffwechsel des Stammskelettes und der proximalen Extremitäten als Ausdruck eines Hyperinflammationssyndroms DD: einer hämatoonkologischen Grunderkrankung (Abb. 3). Ergänzend erfolgte eine erneute Knochenmarkpunktion, bei der das Bild eines toxischen Knochenmarkschadens DD: myelodysplastisches Syndrom mit auffälliger Vakuolisierung der Proerythroblasten sowie der Promyelozyten beschrieben wurde (Abb. 4).

Molekulargentisch wurde ein Missense-Variante c.122T > C; p.Met41Thr mit einem Frequenzanteil von 0,828 im *UBA1-Gen* als hämatopoetisches Mosaik (hemizygot) nachgewiesen, sodass die Diagnose eines VEXAS-Syndroms gestellt werden konnte.

## References

[1] McDermott MF, Aksentijevich I, Galon J, McDermott EM, Ogunkolade BW, Centola M (1999). Germline mutations in the extracellular domains of the 55 kDa TNF receptor, TNFR1, define a family of dominantly inherited autoinflammatory syndromes. Cell.

[2] Loock J (2012). Schnitzler-Syndrom. Z Rheumatol.

[3] Mekinian A, Grignano E, Braun T, Decaux O, Liozon E, Costedoat-Chalumeau N (2016). Systemic inflammatory and autoimmune manifestations associated with myelodysplastic syndromes and chronic myelomonocytic leukaemia: a French multicentre retrospective study. Rheumatology.

[4] Grayson PC, Patel BA, Young NS (2021). VEXAS syndrome. Blood..

[5] Beck DB, Ferrada MA, Sikora KA, Ombrello AK, Collins JC, Pei W (2020). Somatic mutations in UBA1 and severe adult-onset autoinflammatory disease. N Engl J Med.

[6] Poulter JA, Collins JC, Cargo C, De Tute RM, Evans P, Ospina Cardona D (2021). Novel somatic mutations in UBA1 as a cause of VEXAS syndrome. Blood.

[7] Bourbon E, Heiblig M, Gerfaud Valentin M, Barba T, Durel C-A, Lega JC (2021). Therapeutic options in VEXAS syndrome: insights from a retrospective series. Blood.

[8] Shaukat F, Hart M, Burns T, Bansal P (2021). UBA1 and DNMT3A mutations in VEXAS syndrome. A case report and literature review. Mod Rheumatol. Case Reports.

[9] Zakine E, Schell B, Battistella M, Vignon-Pennamen M‑D, Chasset F, Mahévas T (2021). UBA1 variations in neutrophilic dermatosis skin lesions of patients with VEXAS syndrome. JAMA Dermatol.

[10] Dehghan N, Marcon KM, Sedlic T, Beck DB, Dutz JP, Chen LYC (2021). Vacuoles, E1 enzyme, X-linked, autoinflammatory, somatic (VEXAS) syndrome: fevers, myalgia, arthralgia, auricular chondritis, and erythema nodosum. Lancet.

[11] Ferrada MA, Sikora KA, Luo Y, Wells KV, Patel B, Groarke EM (2021). Somatic mutations in UBA1 define a distinct subset of relapsing polychondritis patients with VEXAS syndrome. Arthritis Rheumatol.

[12] Tsuchida N, Kunishita Y, Uchiyama Y, Kirino Y, Enaka M, Yamaguchi Y (2021). Pathogenic UBA1 variants associated with VEXAS syndrome in Japanese patients with relapsing polychondritis. Ann Rheum Dis.

[13] Muratore F, Marvisi C, Castrignanò P, Nicoli D, Farnetti E, Bonanno O (2021). VEXAS syndrome: a case series from a single-center cohort of Italian patients with vasculitis. Arthritis Rheumatol..

[14] Georgin-Lavialle S, Terrier B, Guedon AF, Heiblig M, Comont T, Lazaro E (2021). Further characterization of clinical and laboratory features occurring in VEXAS syndrome in a large-scale analysis of multicenter case-series of 116 French patients. Br J Dermatol.

[15] Gurnari C, Pagliuca S, Durkin L, Terkawi L, Awada H, Kongkiatkamon S (2021). Vacuolization of hematopoietic precursors: an enigma with multiple etiologies. Blood.

[16] Sikora KA, Wells KV, Bolek EC, Jones AI, Grayson PC (2021). Somatic mutations in rheumatological diseases: VEXAS syndrome and beyond. Rheumatology.

[17] Arlet J‑B, Terrier B, Kosmider O (2021). Mutant UBA1 and Severe Adult-Onset Autoinflammatory Disease. N Engl J Med.

[18] Grey A, Cheong PL, Lee FJ, Abadir E, Favaloro J, Yang S (2021). A case of VEXAS syndrome complicated by hemophagocytic lymphohistiocytosis. J Clin Immunol.

[19] Staels F, Betrains A, Woei-A-Jin FJSH, Boeckx N, Beckers M, Bervoets A (2021). Case report: VEXAS syndrome: from mild symptoms to life-threatening macrophage activation syndrome. Front Immunol.

[20] Euvrard R, Fournier T, Georgescu D, Bourbon E, Sujobert P, Lega JC (2021). VEXAS syndrome-related AA amyloidosis: a case report. Rheumatology.

[21] Comont T, Heiblig M, Rivière E, Terriou L, Rossignol J, Bouscary D (2021). Azacitidine for patients with Vacuoles, E1 Enzyme, X‑linked, Autoinflammatory, Somatic syndrome (VEXAS) and myelodysplastic syndrome: data from the French VEXAS registry. Br J Haematol.

[22] Diarra A, Duployez N, Terriou L (2021). Mutant UBA1 and severe adult-onset autoinflammatory disease. N Engl J Med.

